# Effects of *Cistanche deserticola* Y.C. Ma Supplementation on Muscle Strength and Recovery: A Randomized Controlled Trial

**DOI:** 10.3390/nu17182965

**Published:** 2025-09-16

**Authors:** Biaoxu Tao, Weihao Lian, Rongrong Min, Xiaoyu Zhang, Liqi Chen, Sun Hao, Ze Li, Chengzhe Ma, Haojie Zhang, Chang Liu

**Affiliations:** 1 School of Sports Training, Wuhan Sports University, Wuhan 430079, China; 2024410681@whsu.edu.cn (B.T.); 2024410666@whsu.edu.cn (R.M.); 2024410712@whsu.edu.cn (X.Z.); 2024410703@whsu.edu.cn (S.H.); 2 College of Physical Education, Zhengzhou University, Zhengzhou 450001, China; l878585301@163.com; 3 School of Sport Science, Beijing Sport University, Beijing 100084, China; liqi20011004@gmail.com (L.C.); 2024110123@bsu.edu.cn (Z.L.); 4 Faculty of Education, University of Macau, Macao 999078, China; stefanma888@gmail.com

**Keywords:** *Cistanche deserticola* Y.C. Ma, muscle strength, muscle recovery, testosterone, resistance training

## Abstract

Objective: This study aimed to evaluate the effects of *Cistanche deserticola* Y.C. Ma (CD) supplementation on muscle strength and recovery in individuals with and without resistance training experience. Methods: A randomized, double-blind, placebo-controlled trial was conducted with 48 male participants, including 24 resistance-trained and 24 untrained individuals. Participants were stratified by training status and randomly assigned to either the CD or placebo (PLAC) group. All subjects completed a standardized resistance training program three times per week for eight weeks. The CD group received 5 g of CD extract twice daily, while the PLAC group consumed a matched placebo. Assessments included one-repetition maximum (1RM) for bench press and squat, maximal voluntary isometric contraction (MVIC), and repetitions to failure (RTF). Blood biomarkers including serum testosterone, cortisol, C-reactive protein (CRP), and creatine kinase (CK) were also measured. Results: No significant differences in dietary energy intake or macro-nutrient composition were observed based on two 5-day dietary records collected before baseline testing and at the end of the intervention. Among untrained individuals, the CD group showed significantly greater improvements in 1RM bench press and squat compared with the PLAC group (*p* < 0.05), with MVIC and RTF also significantly increased (*p* < 0.01). Serum cortisol levels were reduced (*p* < 0.05), and significant improvements were observed in testosterone, CRP, and CK (*p* < 0.01). In trained individuals, CD supplementation led to significant increases in 1RM squat and MVIC (*p* < 0.05), along with improvements in testosterone and cortisol levels (*p* < 0.05) and marked reductions in CRP and CK (*p* < 0.01). Conclusions: Daily supplementation with 5 g of CD extract for eight consecutive weeks significantly enhanced muscle strength and endurance in males with different training backgrounds and facilitated post-exercise recovery by modulating hormonal responses and reducing stress levels and inflammation. These findings provide experimental evidence supporting the application of CD in sports nutrition.

## 1. Introduction

Modern medical literature and traditional Chinese medicine classics have both documented the multi-system health benefits of *Cistanche deserticola* Y.C. Ma (commonly known as “desert ginseng”) [1,2]. It exerts anti-fatigue, antibacterial, anti-inflammatory, anti-tumor, anti-stress, hypoglycemic, neuroprotective, immunoregulatory, and cardioprotective effects by modulating the nervous, endocrine, cardiopulmonary, energy metabolism, and immune systems [3]. However, compared with its extensive research in cardiovascular, immune, and neuroprotective domains, scientific exploration of CD as an ergogenic aid is still relatively limited, and human evidence supporting its potential effects remains scarce [4].

While modern pharmacological evidence continues to accumulate, traditional medicinal theory provides cultural and historical support for CD’s potential in enhancing physical performance [5,6]. The traditional Chinese medicine classic describes CD as “replenishing the five fatigues and seven injuries, tonifying essence and qi, and promoting lightness of body with prolonged use.” Its traditional functions—such as tonifying kidney yang, nourishing essence and blood, and strengthening tendons and bones—align closely with modern goals of improving athletic performance [7]. From a modern medical perspective, CD, as a representative tonic herb recognized as both food and medicine, possesses dual pharmacological and nutritional attributes. It helps maintain metabolic balance, enhance resistance to external stressors, and support optimal functional status for health and physical performance [8,9]. Since its inclusion in the 2018 official catalogue of substances traditionally used as both food and Chinese medicine, its application has expanded rapidly. As of July 2025, 74 health food products containing CD have been registered in China, primarily targeting fatigue reduction and immune modulation, reflecting its policy support and market potential.

**Figure 1:**
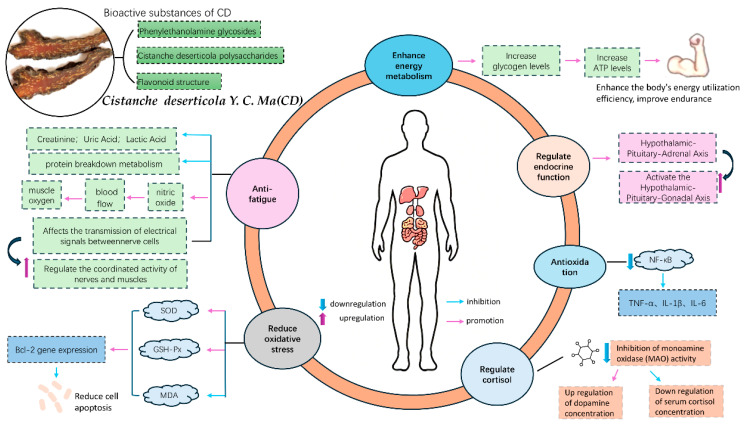
The main pharmacological mechanisms of *Cistanche deserticola* Y.C. Ma and its role in promoting muscle strength and recovery.

Recent systematic studies have comprehensively elucidated the pharmacological properties of CD [1]. Its bioactive compounds have been shown to enhance energy metabolism, improve muscle function, and increase endurance, thereby contributing to overall physical performance [10,11]. These effects are partly achieved by reducing lactate accumulation and anti-stress properties, ensuring more efficient energy utilization. Animal studies further demonstrate that CD constituents elevate adenosine triphosphate (ATP), liver glycogen, and muscle glycogen levels, while reducing creatinine, uric acid, inorganic phosphate, and lactate concentrations, thus delaying the onset of exercise-induced fatigue [12]. In vitro findings indicate that CD can modulate adenosine monophosphate–activated protein kinase (AMPK) activity to maintain energy homeostasis and alleviate oxidative stress. Moreover, the synergistic actions of alkaloids, polysaccharides (CPs), phenylethanoid glycosides (PhGs), and flavonoids provide sustained energy, enhance strength and endurance, and mitigate exercise-related fatigue [13]. These broad physiological benefits are largely attributed to key bioactive constituents, such as phenylethanoid glycosides (e.g., echinacoside, acteoside; Figure 1) and polysaccharides, which possess antioxidant, immunomodulatory, cytoprotective, and anti-stress properties [3,14,15]. Toxicological assessments have confirmed the safety of CD extracts, providing a solid foundation for their dual application in medicinal and nutritional contexts [16].

In exercise physiology, resistance training is considered a key modality for promoting muscle strength and structural remodeling [17]. Adaptations arise primarily from three types of stimuli: mechanical tension, metabolic stress, and muscle fiber microdamage induced by eccentric contractions [18]. Importantly, resistance training per se does not directly enhance muscle strength; rather, the strength gains emerge from recovery and subsequent adaptations following the training stimulus. Resistance exercise induces transient fatigue and muscle damage, acutely impairing performance, but with adequate recovery, these stimuli trigger supercompensation—progressive improvements exceeding the initial capacity [19]. This adaptive process is mediated by delayed yet coordinated biological responses, including satellite cell proliferation, increased myonuclei, and muscle fiber hypertrophy, which typically peak 10–24 days after the cessation of training. At the molecular level, these adaptations involve multiple signaling cascades, with the mTOR/IGF-1 axis and the AMPK/PGC-1α pathway serving as central regulators of myofibrillar protein synthesis, mitochondrial biogenesis, and metabolic remodeling, while satellite cell activation supports repair and long-term strength augmentation [20]. Notably, resistance training protocols performed to failure have been shown to more effectively elicit these supercompensatory responses.

Resistance exercise may induce muscle damage, leading to elevated serum levels of creatine kinase and lactate dehydrogenase, as well as increased lactate accumulation and alterations in blood glucose levels [5,21]. Studies have shown that CD can reduce serum creatine kinase, lactate dehydrogenase, and lactate levels in mice, while increasing hemoglobin and glucose concentrations, which may suggest its potential role in facilitating recovery following resistance exercise [22,23]. In addition to upregulating the IGF-1/PI3K/Akt/mTOR pathway and downregulating atrophy-related ubiquitin ligases (MuRF-1 and MAFbx), phenylethanoid glycosides in CD (e.g., echinacoside, acteoside) appear to engage unique mechanisms not typically associated with conventional ergogenic aids [24,25]: (1) Activation of the PI3K/Akt/GSK3β signaling cascade increases glycogen synthase (GS) expression, thereby promoting mitochondrial DNA replication, oxidative phosphorylation capacity, and glycogen storage, all of which support muscular energy demands [8,26]; (2) Inhibiting monoamine oxidase (MAO) activity, elevating central dopamine concentrations, and lowering serum cortisol levels, thereby regulating neurotransmission, optimizing neuromuscular coordination, alleviating tension and anxiety, and ultimately mitigating fatigue symptoms [5,27]; (3) Modulation of the hypothalamic–pituitary–gonadal (HPG) axis through upregulation of kisspeptin-GPR54 signaling and CYP450–3β-HSD expression facilitates the conversion of cholesterol to steroid hormones, ultimately enhancing testosterone synthesis and supporting muscle protein anabolism and repair [4,26].

Furthermore, CD-derived polysaccharides may reduce secondary muscle injury and NF-κB–mediated inflammatory responses (e.g., C-reactive protein and interleukin-6 expression) by inhibiting NADPH oxidase–driven ROS generation through Nrf2-dependent mechanisms [13,27].

To date, no randomized controlled trial has systematically evaluated the physiological mechanisms and comprehensive effects of CD in resistance training adaptation among individuals with differing training backgrounds. Therefore, this study employed a randomized, double-blind, placebo-controlled design to investigate the effects of an 8-week CD supplementation regimen combined with standardized resistance training on muscle strength, endurance, hormonal responses, stress levels, inflammatory markers, and recovery capacity in both trained and untrained individuals. The findings aim to provide scientific evidence for the translational application of traditional tonic herbs in sports nutrition and promote the integration of CD from traditional medicinal diets into modern exercise health products.

## 2. Materials and Methods

### 2.1. Participants

**Figure 2:**
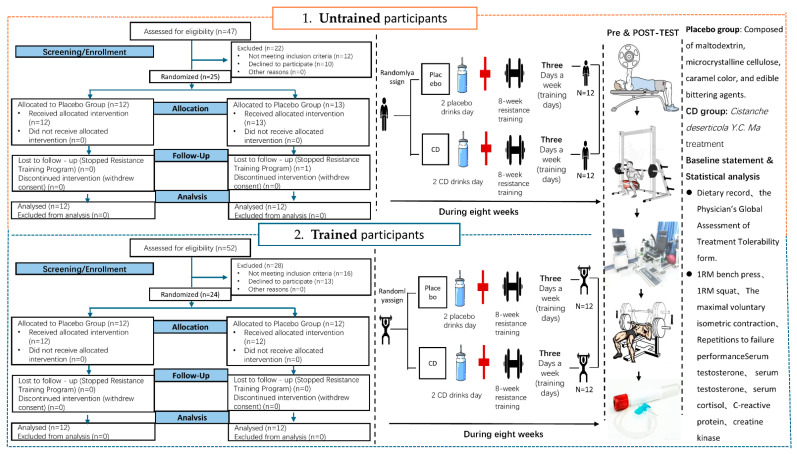
Experimental flowchart.

This study recruited participants from Wuhan Sports University using an age- and BMI-matched design. A total of 48 male participants were enrolled, including 24 resistance training-untrained (RT-untrained) and 24 resistance training-trained (RT-trained) individuals. Within each training status group, participants were assigned to either the placebo (PLAC) group or the CD supplementation group (*n* = 12 per group) using a random sequence generator (https://www.graphpad.com/quickcalcs/randomize1/, accessed on 16 May 2025) with a 1:1 allocation ratio [28]. Allocation concealment was strictly maintained, and both participants and investigators were blinded to group assignments throughout the study (Figure 2). Participants who did not meet the inclusion criteria, refused to participate, or failed to adhere to the resistance training program were excluded from the study.

RT-trained participants were required to meet the following criteria: (a) at least 18 months of consistent resistance training (3–5 sessions per week); (b) ability to perform a 1-repetition maximum (1RM) bench press ≥1.25 × body weight and a full-depth back squat ≥1.75 × body weight; (c) provision of a detailed description of their training regimen; (d) no current or prior use of anabolic-androgenic steroids or performance-enhancing supplements in the last 12 weeks; and (e) agreement to abstain from any additional supplements or over-the-counter compounds that could potentially affect muscle growth or high-intensity training performance during the study period [29]. RT-untrained participants were defined as having no systematic resistance training experience in the previous 12 months and no history of anabolic steroid or supplement use. General exclusion criteria for all participants included the presence of cardiovascular, metabolic, or musculoskeletal disorders.

### 2.2. Ethics Approval

This study was conducted in accordance with the principles of the Declaration of Helsinki and was approved by the Ethics Committee of Wuhan Sports University (Approval No: 2025110).

### 2.3. Study Design

This study employed an 8-week, randomized, double-blind, placebo-controlled, parallel-group design to evaluate the potential effects of CD supplementation on muscle strength and recovery in participants with differing resistance training experience. Adverse events (e.g., gastrointestinal discomfort, headache, dizziness, sleep disturbances, or musculoskeletal complaints) were assessed through participant/researcher reports and the Physician’s Global Assessment of Treatment Tolerability (PGATT) form [30].

### 2.4. Supplementation Preparation and Management

*Cistanche deserticola* Y.C. Ma (CD) was cultivated in Inner Mongolia, and the raw material was provided by Beijing Tongrentang (Hohhot Shancheng Biotechnology Co., Ltd., Hohhot, China) for extract preparation. The extraction procedure was as follows: the dried CD was coarsely powdered while retaining the scales and leaves and extracted three times with 60% ethanol in a 70 °C water bath (2 h each, material-to-solvent ratio 1:8) [1]. The combined extracts were refrigerated at 4 °C for 24 h and then filtered. The filtrate was subjected to vacuum ethanol recovery at −0.08 MPa and 50 °C, followed by concentration under the same vacuum conditions (−0.08 MPa, 50 °C) to a drug-to-extract ratio (DER, native) of 2:1. The resulting concentrate was transferred to a vacuum drying chamber (−0.08 MPa, 65 °C) and dried to a moisture content ≤5%, yielding the dry extract [23,31,32]. The daily dosage was determined based on the *Encyclopedia of Traditional Chinese Medicine* (2nd edition, Volume I), which recommends a 10 g daily intake of raw CD. This corresponds to a dosage of 5 g extract per day [3]. The placebo was composed of maltodextrin, microcrystalline cellulose, caramel coloring, and a food-grade bittering agent, and was matched to the CD extract in appearance, weight, and texture [33]. All samples were individually sealed in light-resistant aluminum foil sachets and stored under dark, dry, and moisture-proof conditions. Participants were instructed to consume one sachet twice daily, following breakfast and lunch [4].

### 2.5. Training Intervention Program

This study implemented an 8-week resistance training program designed to systematically enhance participants’ muscle strength [34]. Training frequency was set at three sessions per week, each targeting specific muscle groups including the chest, back, legs, and shoulder-arm muscles. Each session comprised 4–5 exercises, following the professional guidelines published by the National Strength and Conditioning Association (NSCA). All training was conducted within the same fitness facility using standardized brand equipment to ensure consistent experimental conditions. A 5 min low-intensity aerobic warm-up was performed before each session. The training process was fully supervised by an experienced coaching team, with each coach responsible for overseeing three participants to ensure training quality and safety [35].

During the initial four weeks, participants completed 4 sets of 10–12 repetitions at 65–70% of their 1RM. In the subsequent four weeks, the training protocol progressed to 4 sets of 6–8 repetitions at 80% of 1RM. This progression aimed to gradually increase training load and promote muscular adaptations [36].

### 2.6. Daily Dietary Intake Recording

To control for the potential impact of dietary factors on study outcomes, participants were required to maintain their habitual diets and were prohibited from using any nutritional supplements other than the study intervention. Dietary adherence was monitored using a digital dietary recording method. Participants completed two 5-day dietary records using the professional nutrition analysis software MyFitnessPal (Version 25.35): the first record was collected one week prior to baseline testing, and the second at the end of the intervention [37]. The research team provided standardized training to instruct participants on accurately documenting food types, intake amounts, and portion sizes. This analysis system automatically generated key nutritional parameters, including daily total energy intake and macronutrient energy distribution.

### 2.7. Baseline Statement

#### 2.7.1. Bench Press/Deep Squat 1RM

The one-repetition maximum (1RM) test, defined as the maximum load an individual can lift once through a full range of motion with proper technique, is a widely accepted method for evaluating absolute muscular strength. In this study, 1RM assessments for upper and lower body strength were conducted using the Smith machine bench press and free-weight back squat, respectively, both at baseline and within 48 h following the intervention [35,38]. All tests were supervised by certified personnel to ensure strict adherence to the technique guidelines recommended by the NSCA. Specifically, the bench press required maintenance of five-point contact (both feet, buttocks, scapulae, and head), with the barbell lowered to the chest and the elbows fully extended upon completion. For the squat, participants were instructed to descend until their thighs were parallel to the ground.

Given the varying resistance training experience among participants—including both novices and individuals with structured training backgrounds—standardized instruction and testing procedures were provided to ensure consistent administration and participant safety [29]. The 1RM testing protocol included the following three stages: (1) Initial warm-up: Participants without training experience performed 10–15 repetitions at a light load under the guidance of certified trainers (starting loads approximately 20 ± 2.5 kg for bench press and 30 ± 2.5 kg for squat). Participants with prior training experience selected an appropriate starting load based on their capacity. (2) Progressive warm-up sets: The load was increased by 10–15% for 6–8 repetitions, followed by a 2–5 min rest. A further 5–10% increase was applied for 3–4 repetitions. This phase aimed to activate neuromuscular function rather than test maximal effort. (3) 1RM attempts: After a 2–5 min rest, participants began formal testing with 5% load increments. Each attempt consisted of 1–2 repetitions; if successful, participants rested and continued until failure. No more than five maximal attempts were allowed per exercise. Rest periods between different loads were set at 3 min, with a 4 min rest between exercises. The heaviest load lifted with correct form was recorded as the final 1RM value.

#### 2.7.2. MVIC Test for the Quadriceps

The maximal voluntary isometric contraction (MVIC) torque of the knee extensors was measured using an isokinetic dynamometer (Biodex System, Biodex Medical Systems, Shirley, NY, USA). During the test, participants maintained a knee flexion angle of 75°, and each maximal isometric contraction was sustained for 5 s [39]. Three consecutive trials were performed, with a 60 s rest interval between trials. The system sampling frequency was set at 2000 Hz, and the torque measurement accuracy was controlled within ±1%.

#### 2.7.3. Repetitions to Failure (RTF) Performance

Upper body muscular endurance was assessed using a progressive bench press protocol. During the first 4 weeks, participants performed 4 sets of 10–12 repetitions at 60% of their 1RM, a widely applied method in resistance training research. In the subsequent 4 weeks, the protocol progressed to 4 sets of 6–8 repetitions at 70% of 1RM. This staged approach was designed to gradually increase the training load and elicit adaptive muscular responses. The test was supervised by a certified coach to ensure correct technique and consistent tempo. Fatigue was defined by the occurrence of either (1) a pause in movement exceeding 10 s or a marked deviation in posture, or (2) voluntary termination by the participant. Body mass, exercise duration, and the number of valid repetitions were recorded for each participant, with the total number of valid repetitions serving as the primary indicator of muscular endurance [40].

#### 2.7.4. Muscle Recovery

Muscle recovery was evaluated through a comprehensive analysis of multiple serum biochemical markers. All blood samples were collected following standardized procedures on the morning of day 2 after the start of the study and day 2 after the end of the study under resting conditions [41]. Blood was drawn using vacuum tubes containing clot activator (SST; Becton Dickinson, Oxford, UK; 7 mL), centrifuged (1500× *g*, 10 min) to separate serum, aliquoted, and stored at −80 °C until analysis. Serum testosterone (reference range: 300–1000 ng/dL) and cortisol (reference range: 5–25 μg/dL) levels were measured using commercially available enzyme-linked immunosorbent assay (ELISA) kits (DRG Diagnostics, Marburg, Germany), reflecting anabolic status and stress response, respectively, with both serving as sensitive indicators of recovery capacity. C-reactive protein (CRP, reference value: <5 mg/L) concentrations were determined by immunoturbidimetry (Randox Ltd., Antrim, UK; sensitivity <0.08 mg/L) using an automated clinical chemistry analyzer (RX Daytona^TM^, Furuno, Nishinomiya, Japan) to assess inflammation and tissue repair processes. Creatine kinase (CK, reference range: 50–310 U/L) activity was quantified using VITROS CK slides (Ortho Clinical Diagnostics, Buckinghamshire, UK) and analyzed on the VITROS 950 chemistry system, with its level measured on the morning following exercise serving as an important indicator of muscle damage and recovery status. Collectively, these biomarkers constituted a multidimensional evaluation system for muscle recovery, with their patterns of change providing sensitive insights into training load and adaptation [42,43].

### 2.8. Tolerability

Adverse events were monitored using a standardized reporting procedure whereby participants were required to actively report any discomfort experienced throughout the study. Tolerability was assessed using the clinically validated Physician’s Global Assessment of Treatment Tolerability (PGATT) scale, which employs a 5-point rating system ranging from 1 (extremely intolerable) to 5 (completely tolerable). This scale provides an objective measure of participants’ tolerance to the intervention, with a score of 5 indicating no adverse effects and excellent tolerability [44].

### 2.9. Statistical Analysis

Results are expressed as mean ± standard deviation (SD). Baseline differences were assessed using independent *t*-tests. Repeated measures analysis of variance (ANOVA) was employed to evaluate the statistical significance of treatment effects over time, with group assignment (treatment vs. placebo) as the between-subject factor. For data that did not meet the assumption of normality, the Mann–Whitney test was applied. Change scores were calculated using incremental values, and between-group differences were expressed as the mean difference with 95% confidence intervals (CIs). Statistical significance was set at *p* < 0.05 (*), *p* < 0.01 (**), and *p* < 0.001 (***); “ns” indicates no statistical difference. All analyses were performed using Origin 2025 and SPSS version 26.0 (IBM Corp., Armonk, NY, USA) [45].

## 3. Results

### 3.1. Dietary Intake Records

To minimize potential confounding from dietary variables, strict standardized dietary monitoring was implemented for all participants, including both untrained and resistance-trained participants. Baseline nutritional intake was comparable between the placebo (PLAC) and Cistanche deserticola (CD) groups in both cohorts, with no statistically significant differences (*p* > 0.05).

As shown in Table 1, dietary patterns remained consistent throughout the study. Trained participants consumed more total energy than untrained participants, mainly due to higher carbohydrate intake, while protein and fat intake differed to a lesser extent. Despite these expected differences by training status, no significant differences were observed between experimental groups at any time point (*p* > 0.05), indicating that the dietary control strategy effectively minimized nutrition-related confounding.

### 3.2. Changes in Untrained Participants After Supplementation with Training Supplements

#### 3.2.1. Muscle Strength

After 8 weeks of resistance training, both the CD and PLAC groups exhibited significant improvements in upper- and lower-limb muscle strength and endurance, consistent with the expected effects of training intervention. The primary aim of this study, however, was to determine whether CD supplementation could elicit superior adaptive gains.

**Figure 3:**
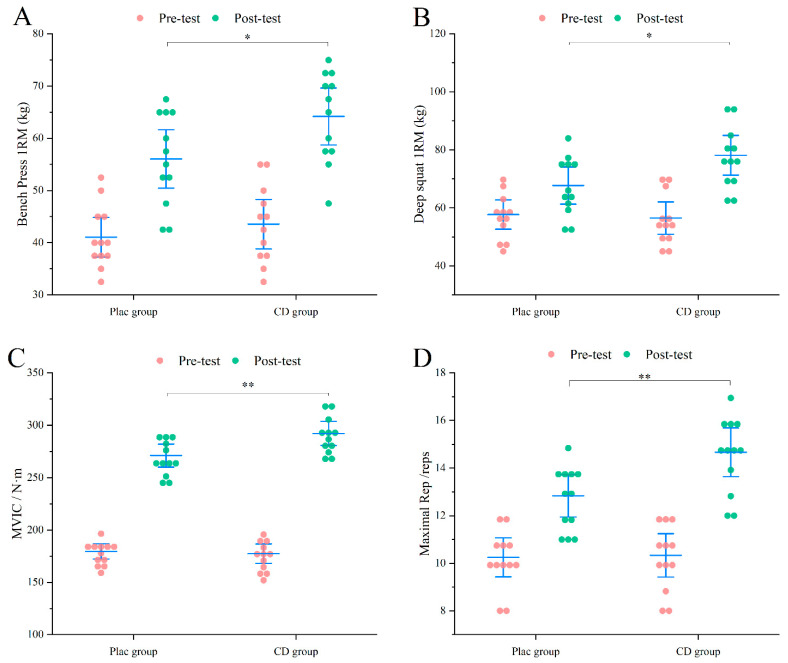
Exercise performance results of untrained participants. (**A**) Bench press 1RM. (**B**) Deep squat 1RM. (**C**) MVIC. (**D**) RTF performance. * *p* < 0.05, ** *p* < 0.01.

As shown in Figure 3, in the PLAC group, 1RM bench press was 56.04 ± 8.82 kg, 1RM squat was 67.70 ± 10.08 kg, MVIC was 272.93 ± 17.48 N·m, and RTF at 60% 1RM was 13 ± 1. In contrast, all performance indicators in the CD group were significantly higher (*p* < 0.05) (Figure 3): 1RM bench press increased to 64.16 ± 8.55 kg, 1RM squat reached 78.12 ± 10.77 kg, MVIC improved to 291.82 ± 19.35 N·m (*p* < 0.01), and RTF rose to 15 ± 2 repetitions (*p* < 0.01). These findings indicate that CD supplementation led to markedly greater improvements in both muscle strength and endurance compared with placebo, suggesting a potential ergogenic benefit when combined with resistance training.

#### 3.2.2. Muscle Recovery

**Figure 4:**
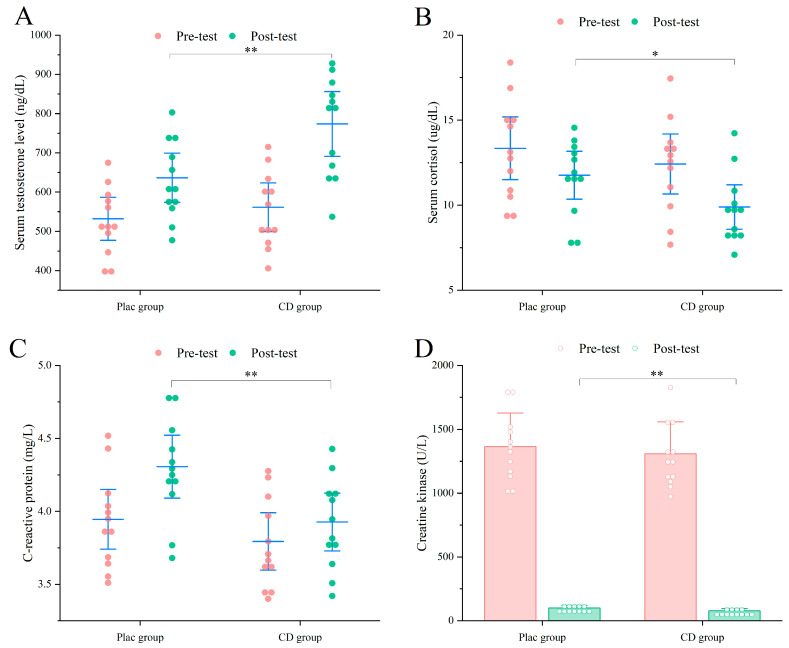
Exercise performance results of untrained participants. (**A**) Serum Testosterone. (**B**) Serum cortisol. (**C**) CRP. (D) CK. * *p* < 0.05, ** *p* < 0.01.

Test results demonstrated that serum testosterone levels increased significantly more in the CD group compared with the PLAC group (PLAC: 636.49 ± 98.85 ng/dL vs. CD: 773.77 ± 130.01 ng/dL; *p* < 0.01) (Figure 4). In addition, the CD group showed a greater reduction in serum cortisol (PLAC: 11.76 ± 2.21 µg/dL vs. CD: 9.89 ± 2.05 µg/dL; *p* < 0.05). Serum CRP levels were also markedly lower in the CD group (PLAC: 4.30 ± 0.33 mg/L vs. CD: 3.92 ± 0.31 mg/L; *p* < 0.01), along with a significant decrease in CK activity (PLAC: 100.39 ± 19.86 U/L vs. CD: 78.33 ± 16.32 U/L; *p* < 0.01). Collectively, these changes suggest that CD supplementation may promote post-resistance training muscle repair and recovery by enhancing anabolic hormone levels, reducing catabolic and stress-related hormones, and attenuating inflammatory and muscle damage biomarkers.

### 3.3. Changes in Trained Participants After Supplementation with Training Supplements

#### 3.3.1. Muscle Strength

**Figure 5:**
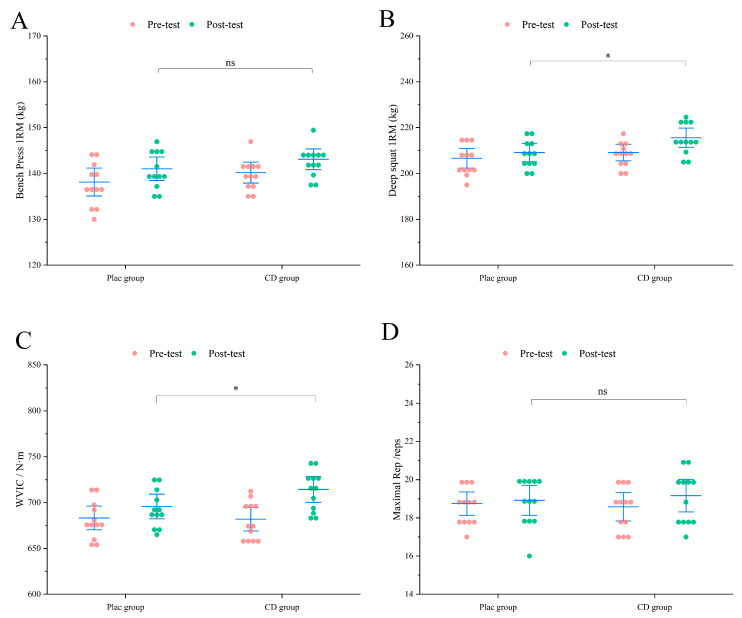
Exercise performance results of trained participants. (**A**) Bench press 1RM. (**B**) Deep squat 1RM. (**C**) MVIC. (**D**) RTF performance. * *p* < 0.05, ns = not significant (*p* ≥ 0.05).

Test results indicated that, although the CD group showed numerically higher values than the PLAC group in 1RM bench press (PLAC: 141.04 ± 4.05 kg vs. CD: 143.12 ± 3.55 kg) and 60% 1RM maximal repetitions (PLAC: 18.91 ± 1.24 reps vs. CD: 19.16 ± 1.33 reps), these differences did not reach statistical significance (*p* > 0.05) (Figure 5). In contrast, significant improvements were observed in 1RM squat (PLAC: 209.16 ± 6.24 kg vs. CD: 215.62 ± 6.66 kg; *p* < 0.05) and MVIC (PLAC: 695.83 ± 21.09 N·m vs. CD: 714.41 ± 22.30 N·m; *p* < 0.05), indicating that CD supplementation significantly enhanced lower-limb strength and maximal isometric torque compared with PLAC.

#### 3.3.2. Muscle Recovery

**Figure 6:**
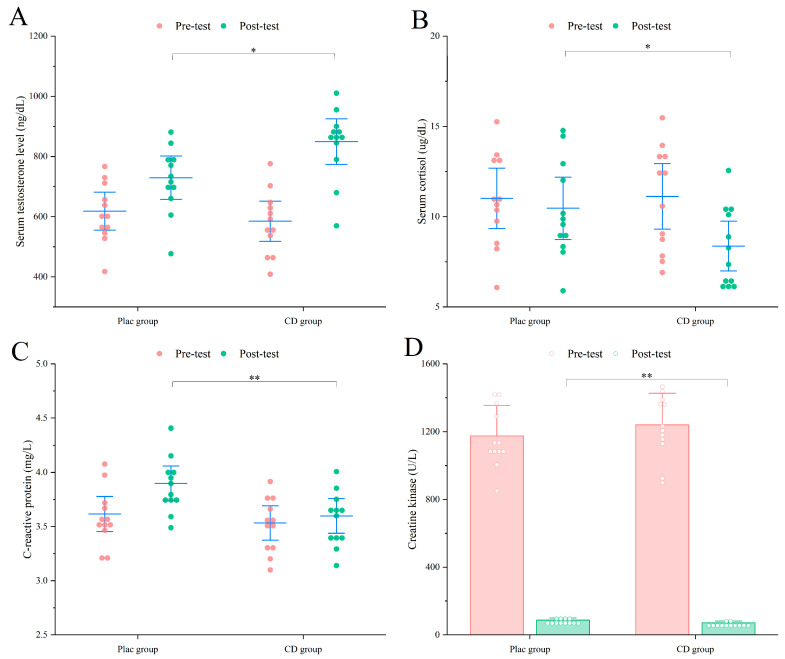
Exercise performance results of trained participants. (**A**) Serum Testosterone. (**B**) Serum cortisol. (**C**) CRP. (**D**) CK. * *p* < 0.05, ** *p* < 0.01.

Following the resistance training program, post-intervention serum testosterone levels in the PLAC group were 732.16 ± 117.27 ng/dL, serum cortisol was 10.46 ± 2.72 µg/dL, CRP was 3.89 ± 0.25 mg/L, and CK activity was 86.67 ± 13.48 U/L. In contrast, the CD group demonstrated significantly more favorable outcomes across all parameters (*p* < 0.05) (Figure 6): serum testosterone increased to 846.15 ± 141.26 ng/dL, cortisol decreased to 8.32 ± 2.17 µg/dL, and both CRP (3.59 ± 0.25 mg/L) and CK (71.46 ± 10.68 U/L) were further reduced (*p* < 0.01). These findings indicate that even in resistance-trained individuals, 8 weeks of continuous CD supplementation can significantly enhance post-resistance training recovery.

## 4. Discussion

This study aimed to evaluate the effects of CD supplementation on muscle strength and recovery in individuals with and without prior resistance training experience. For the first time, we demonstrated that CD supplementation significantly enhanced muscle strength, endurance, and recovery capacity in untrained individuals. Among trained participants, improvements were primarily observed in lower limb maximal strength and recovery indicators. These findings suggest that CD supplementation is not only effective for novices but also beneficial for experienced individuals. To ensure the reliability of the results, potential confounding factors—including training intensity, training history, dietary intake, and sleep—were strictly controlled. Specifically, participants were instructed to maintain a regular sleep schedule of approximately 7–8 h per night. This ensured that the observed improvements could be attributed to the supplementation, thereby supporting our initial hypothesis [30].

All participants showed good tolerance throughout the intervention and the one-month post-trial period, with no adverse events reported. The observed improvements in muscle strength and endurance are consistent with previous studies [6,8,9]. The active constituents in CD, such as phenylethanoid glycosides and oligosaccharides, may enhance muscle strength by modulating energy metabolism pathways and key enzymatic activities [46]. Specifically, CD activates the AMPK signaling pathway, promotes the expression and membrane translocation of glucose transporters (e.g., GLUT4), and upregulates glycolytic enzymes like phosphofructokinase (PFK) and pyruvate kinase (PK), thereby accelerating glycolysis and facilitating rapid ATP supply for muscle contraction [47,48]. Furthermore, CD polysaccharides may enhance the activity of succinate dehydrogenase (SDH), a key rate-limiting enzyme in the TCA cycle, thereby improving mitochondrial oxidative metabolism and ATP production [49]. CD also appears to inhibit mitochondrial membrane potential collapse and cytochrome c release, reducing apoptosis and preserving the structural foundation of muscle contraction [50]. Additionally, phenylethanoid glycosides may downregulate aberrant creatine kinase (CK) expression in muscle cells, reducing muscle damage biomarkers and protecting fiber integrity [51], with serum CK levels reflecting post-exercise muscle damage and recovery status.

Regarding fatigue alleviation induced by resistance training, previous studies have reported that CD may improve cardiovascular function, enhance antioxidant defenses, and exert neuromodulatory effects [52]. In terms of vascular function, the bioactive compounds of CD can promote nitric oxide (NO) synthesis and release, thereby improving endothelial function and blood flow, reducing lactate accumulation, lowering plasma viscosity and erythrocyte aggregation, and decreasing thrombotic risk, ultimately enhancing oxygen and nutrient delivery to muscle tissue and alleviating fatigue [53]. In terms of antioxidant activity, CD polyphenols can activate the Nrf2/HO-1 pathway, upregulate antioxidant enzymes such as superoxide dismutase (SOD) and glutathione peroxidase (GPx), and inhibit NADPH oxidase 2 (NOX2) activity, thus reducing reactive oxygen species (ROS) generation and lipid peroxidation (e.g., malondialdehyde [MDA]), ultimately protecting muscle membranes [54]. These effects may contribute to reduced exercise-induced inflammation, reflected in decreased serum CRP levels, thereby facilitating muscle recovery and tissue repair. Neurologically, CD oligosaccharides may inhibit monoamine oxidase (MAO) activity, maintain cerebral dopamine levels, and attenuate hypothalamic–pituitary–adrenal (HPA) axis hyperactivation, thereby reducing cortisol elevation and improving neurotransmitter balance related to central fatigue [55,56]. Serum testosterone serves as a sensitive indicator of muscle anabolic and recovery capacity, whereas serum cortisol reflects stress status and central fatigue regulation. Collectively, these mechanisms contribute to CD’s potential to enhance performance and alleviate fatigue during resistance training [57].

Resistance training is often accompanied by hormonal fluctuations, oxidative stress, inflammation, and muscle damage. An individual’s ability to return to homeostasis post-exercise reflects recovery capacity and determines the sustainability of subsequent training loads. In this study, CD supplementation significantly elevated serum testosterone, reduced oxidative stress and systemic inflammation, and accelerated muscle repair, regardless of training experience [58,59]. These findings suggest that CD facilitates post-training recovery via multiple mechanisms. Previous research has indicated that CD may modulate testosterone production through activation of the hypothalamic–pituitary–gonadal (HPG) axis. Phenylethanoid glycosides may stimulate luteinizing hormone (LH) secretion and enhance cholesterol conversion to testosterone in Leydig cells, potentially via upregulation of steroidogenic acute regulatory protein (StAR) and cytochrome P450 side-chain cleavage enzyme (CYP11A1) [60]. Meanwhile, CD polysaccharides may improve testicular oxidative status, protect spermatogenic and Leydig cell function, and thereby support stable testosterone output necessary for muscle maintenance and recovery [61].

In addition to testosterone, CD exhibited regulatory effects on cortisol, C-reactive protein (CRP), and CK, likely mediated by its anti-stress, anti-inflammatory, and neuroendocrine properties. Regarding cortisol, CD oligosaccharides may suppress excessive HPA axis activation by downregulating corticotropin-releasing hormone (CRH) and adrenocorticotropic hormone (ACTH), thus alleviating catabolic responses to chronic stress [58,62]. Phenylethanoid glycosides and lignans may inhibit NF-κB signaling and reduce proinflammatory cytokine (e.g., TNF-α, IL-6) production, thereby decreasing hepatic CRP synthesis and mitigating systemic inflammation [59]. The modulation of CK levels may involve enhanced antioxidant enzyme activities (e.g., SOD, GPx), which protect muscle membranes from ROS-induced damage and limit CK leakage due to membrane permeability changes [13]. Together, these effects support CD’s integrative role in post-exercise recovery by modulating hormones, mitigating stress and inflammatory responses, and preserving muscle tissue integrity.

This study has several notable strengths. First, the 8-week CD supplementation combined with resistance training represents the first and longest human trial investigating CD in this context. Second, the sample population spanned both untrained individuals and trained athletes, enabling a comprehensive assessment of CD’s effects across varying training statuses—an important factor, as training background significantly influences adaptation to resistance and other exercise forms. Third, the randomized, double-blind, placebo-controlled design—considered the gold standard in clinical research—ensured scientific rigor and reproducibility.

However, this study also has limitations. Although all participants were instructed to follow standardized pre-exercise rest and fasting protocols, adherence may have varied, potentially affecting certain performance metrics. In addition, while muscle hypertrophy was not the primary outcome, the participants’ total energy (23.5–25 kcal/kg/d) and protein intake (1.2–1.3 g/kg/d), although adequate, fell short of current recommendations for optimizing resistance training adaptations. Moreover, the lack of body composition data (e.g., lean mass, fat mass) may have limited a comprehensive interpretation of CD’s effects [63]. Future research should better control dietary variables—possibly through appetite monitoring—and incorporate neuromuscular assessments (e.g., motor unit recruitment and neural activation) to elucidate strength gains more precisely, thereby building a stronger evidence base for CD’s use in sports nutrition.

In conclusion, daily supplementation of 5 g CD extract for 8 consecutive weeks, in combination with progressive high-intensity resistance training, significantly improved upper and lower limb maximal strength and multiple indicators of muscle recovery. Importantly, only the CD group showed statistically significant improvements in 1RM squat strength, MVIC, serum testosterone, cortisol, CRP, and CK levels, suggesting that CD supplementation has a beneficial role in both strength development and post-exercise recovery.

## 5. Conclusions

This study demonstrates that daily supplementation with 5 g of CD extract for 8 consecutive weeks significantly enhances muscle strength and endurance in both untrained and resistance-trained individuals while also improving testosterone levels, alleviating stress levels, reducing inflammation, and promoting muscle recovery.

## Figures and Tables

**Table 1:** Dietary intake data.

					Between-Group Comparison	
Training Status	Variables	*n*	Baseline (Week 0)	Post (Week 8)	95% CI	*p*-Value
Untrained Participants	Calorie Intake (kcals/day)					
	CD	12	2138 ± 611	2339 ± 623	(−5.9, 144.1)	0.76
	PLAC	12	2289 ± 465	2270 ± 504		
	Carbohydrate Intake (g/day)					
	CD	12	210 ± 90	215 ± 94	(−16.6, 3.3)	0.85
	PLAC	12	216 ± 78	222 ± 79		
	Protein Intake (g/day)					
	CD	12	96 ± 35	98 ± 40	(3.7, 8.5)	0.70
	PLAC	12	89 ± 30	92 ± 36		
	Fat Intake (g/day)					
	CD	12	76 ± 31	80 ± 35	(0.1, 13.0)	0.71
	PLAC	12	71 ± 29.92	75 ± 32		
Trained Participants	Calorie Intake (kcals/day)					
	CD	12	3382 ± 651	3578 ± 757	(48.3, 72.6)	0.84
	PLAC	12	3441 ± 691	3518 ± 738		
	Carbohydrate Intake (g/day)					
	CD	12	515 ± 108	557 ± 127	(8.7, 20.1)	0.77
	PLAC	12	520 ± 103	543 ± 118		
	Protein Intake (g/day)					
	CD	12	71 ± 14	73 ± 15	(1.8, 3.3)	0.79
	PLAC	12	68 ± 13	72 ± 14		
	Fat Intake (g/day)					
	CD	12	102 ± 18	109 ± 23	(0.4, 1.0)	0.90
	PLAC	12	103 ± 19	106 ± 22		

## Data Availability

The original contributions presented in this study are included in this article. Further inquiries can be directed to the corresponding authors.
